# Time to Effective Ventilation in Neonatal Manikins with a Supraglottic Airway vs. a Facemask: A Randomized Controlled Trial

**DOI:** 10.3390/children10030498

**Published:** 2023-03-02

**Authors:** Nithya Sivakumar, Shoshana Newman-Lindsay, Deepika Sankaran, Satyan Lakshminrusimha, Lee Donohue

**Affiliations:** Davis Department of Pediatrics, University of California, Sacramento, CA 95817, USA

**Keywords:** neonatal resuscitation, laryngeal mask airway, facemask, positive pressure ventilation, T-piece resuscitator

## Abstract

(1) Background: Timely and effective positive pressure ventilation (PPV) is the most important component of neonatal resuscitation. Emerging data supports the use of supraglottic airways such as the laryngeal mask airway (LMA) as a first-line interface for PPV during neonatal resuscitation. LMA use reduces the need for intubation compared to facemask use in systematic reviews, but there is no difference in the incidence of death or moderate-to-severe hypoxic ischemic encephalopathy (HIE). Time to effective ventilation during simulation with manikin models by providers with limited neonatal airway experience may add to the current evidence that compares the LMA to the neonatal facemask as the first-line ventilation interface during neonatal resuscitation.; (2) Methods: Thirty-two pre-clinical medical students were recruited and randomized to learning and performing ventilation with either the LMA or neonatal facemask on a neonatal manikin. Tidal volume was measured by breath-by-breath analysis to assess adequacy and consistency of PPV in 10 consecutive breaths. Perceived confidence was measured by pre- and post-intervention surveys that utilized a Likert scale from 1 to 5.; (3) Results: Median time to achieve effective ventilation was shorter with a neonatal facemask compared to the LMA (43 (30, 112) seconds vs. 82 (61, 264) seconds, *p* < 0.01). Participants reported higher perceived confidence post-intervention with use of a facemask when compared to use of the LMA (5 (4, 5) vs. 4 (4, 4), *p* = 0.03).; (4) Conclusions: Pre-clinical medical students demonstrated a shorter time to effective ventilation and reported higher confidence scores after learning and demonstrating PPV using the facemask when compared to LMA in a neonatal manikin. Further studies are warranted to evaluate the use of supraglottic airways in providers with limited experience with airway management of neonates, as well as in ways to better promote proficiency and confidence in the use of the LMA.

## 1. Introduction

Positive pressure ventilation (PPV) is the most important component of neonatal resuscitation [1,2,3]. In newborns who require assistance to breathe at birth, it is of utmost importance to provide effective PPV in an efficient and timely manner. Currently, the American Academy of Pediatrics Textbook of Neonatal Resuscitation recommends initiating PPV using a neonatal facemask [2]. If PPV is ineffective despite corrective measures, it is recommended to then switch the ventilation interface to an endotracheal tube (ETT) or supraglottic airway such as the laryngeal mask airway (LMA). However, there is growing interest and evidence to support the use of the LMA as a first-line interface for PPV [4,5,6,7,8,9,10,11,12,13,14,15,16]. 

A 2018 Cochrane review evaluated five trials comparing the LMA to facemask ventilation and observed with low to moderate quality evidence that LMA use reduced intubation rates [6]. More recent systematic [15] and narrative [16] reviews reached similar conclusions after inclusion of Pejovic et al.’s recent large trial [17], which included over 1100 neonates. In two other meta-analyses, it was similarly concluded that the use of supraglottic airways, including the LMA, in the neonatal population was associated with less failure of PPV than use of the facemask and subsequently decreased ETT requirements [15,16]. Diggikar et al. additionally concluded that in low-to-middle income countries, there is no difference in death or moderate-to-severe hypoxic ischemic encephalopathy (HIE) in infants that were given PPV using a neonatal facemask or the LMA [15]. In a randomized controlled trial conducted in Uganda among experienced midwifes, there was no difference in the outcome of death within 7 days or admission to a neonatal intensive care unit (NICU) with moderate-to-severe HIE with the initial use of the LMA versus a facemask as the primary means of providing PPV [17]. 

Many providers attending deliveries in low-resource settings and emergency medical personnel have limited experience providing neonatal airway support, and only a small percentage have used an LMA rather than facemasks. Providers attending deliveries at community hospitals in which there are only a few births per year with less frequent need for PPV will have less experience with providing PPV. Although the number of babies requiring resuscitation is low, the ability to provide effective PPV remains essential to the successful resuscitation of those few babies per year. 

We hypothesized that time to effective ventilation of a neonatal manikin will be shorter with LMA compared to facemask due to the high risk for facemask leaks [18]. Our goal was to generate information about first-line PPV methods for deliveries lacking personnel with extensive neonatal resuscitation experience. Additionally, we evaluated the confidence of unskilled providers in performing adequate ventilation on a neonatal manikin with either an LMA or neonatal facemask and collected subjective information about their ease of use during neonatal resuscitation. 

## 2. Materials and Methods

This study was a manikin-based study conducted with the approval of the University of California Davis Institutional Review Board (IRB 1722704-1). The study was conducted at the UC Davis Center for Simulation and Education in the School of Medicine. We used the Gaumard neonatal manikin model (Code Blue III Newborn Resuscitation and Emergency Simulator; Gaumard, Miami, FL, USA) and the associated software to measure the study endpoints. We used a size 1 inflatable cuff LMA (Teleflex Incorporated, Wayne, PA, USA) and a standard neonatal face mask, both with a Neo-Tee Infant T-piece resuscitator (Mercury Medical, Clearwater, FL, USA). 

### 2.1. Participants

We chose medical students as a surrogate for providers with limited neonatal airway experience, such as those working in community hospitals. The participants for this study were pre-clinical medical students from the UC Davis School of Medicine. Pre-clinical medical students were screened for eligibility prior to recruitment by the first author (NS). All medical students had active certification in Basic Life Support, and students with prior experience with LMA placement were excluded. Thirty-two medical students were randomized to learning and then performing PPV using either an LMA or a neonatal facemask with a T-piece resuscitator. 

### 2.2. Study Design

We conducted a randomized trial comparing the LMA and neonatal facemask in a neonatal manikin model. Participants were randomized to PPV method by a random-number generator. Sessions included only participants assigned to the same PPV method and included 2–3 participants who were not blinded to the intervention. Participants learned, practiced, and then performed PPV using either the LMA or neonatal facemask. Pre- and post-intervention surveys were given to each participant to measure confidence and ability prior to and after the study visit, respectively. Each participant was compensated for their time with a $10 gift card. A flow diagram illustrating this study design can be found in the Supplemental Materials (Supplement S1).

### 2.3. Sample Size

Our sample size was calculated for the primary outcome based on our preliminary research. If the true difference in time to effective ventilation using the neonatal facemask versus LMA is 45 s, with a standard deviation of 30 s, we would need to study 16 subjects in each group to be able to reject the null hypothesis that the time to achieve effective ventilation in the LMA and neonatal face mask groups are equal with a probability power of 0.9. The Type I error probability associated with this null hypothesis is 0.05.

### 2.4. Conduct of Experiment

The participants completed a pre-visit survey (Supplement S2) and then were provided with educational modules for neonatal resuscitation with their assigned device. The video modules shown were from the Neonatal Resuscitation Program (NRP). The initial video was common for the LMA and neonatal face mask groups, from https://www.youtube.com/watch?v=72ngAsVmD5w (accessed on 1 January 2022). The second video was specific for the group (participants randomized to the LMA viewing a clip from https://www.youtube.com/watch?v=TPBQd35EVgk&t=4s (accessed on 1 January 2022). and participants randomized to the facemask viewing a clip from https://www.youtube.com/watch?v=aAlMreEBKYU (accessed on 1 January 2022). A live demonstration was then performed by an NRP instructor with a standardized script. Participants were taught to perform two-person PPV. We provided a training similar to the airway portion of NRP training utilizing either the neonatal facemask or LMA. We then asked them to conduct the airway portion of a neonatal resuscitation. In the facemask group, one person placed, positioned, and held the face mask to maintain a good seal and positioned the airway. The second person provided breaths. In the LMA group, one person would place, position, and inflate the cuff of the LMA, and the second person provided breaths. All participants practiced both roles prior to the start of the timed trial. Participants were encouraged to ask questions and seek guidance. At the conclusion of the testing visit, all participants were asked to complete a post-visit survey (Supplement S3).

### 2.5. Testing of Participants

**Figure 1:**
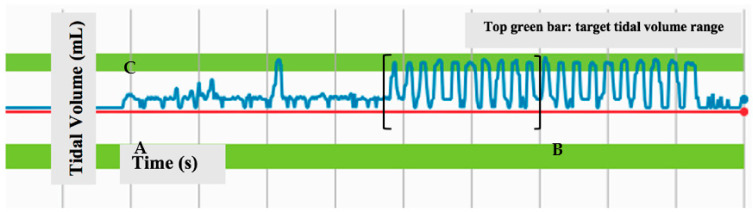
Screenshot of Gaumard software depicting consecutive breaths that meet target tidal volumes. Green bar indicates target tidal volume range, and brackets show ten consecutive breaths of adequate tidal volume. Point A depicts the point at which timing of the trial began, and Point B is the time at which the trial ended. The labeled bar C represents the range specified to be the range at which a breath was deemed “adequate”.

After some hands-on practice with the manikins (approximately 20 min), effectiveness of ventilation was assessed. Time to effective ventilation was defined as the time from when the assigned device was picked up to the time they achieved 10 consecutive, adequate breaths provided to the neonatal manikin as measured by the Gaumard software. An example of the Gaumard software output is shown in Figure 1, where point A depicts the moment at which the participant picked up their assigned device and point B depicts the moment at which the participant achieved 10 consecutive, adequate breaths. Time to effective ventilation was defined as the time between point A and point B. An adequate breath was defined as a breath that reached the goal tidal volume (green bar, C, Figure 1) from the baseline. Goal tidal volume was determined by pre-setting the device with breaths that were administered by experienced NRP instructors, prior to any trials with our participants. Each participant was timed twice during the study for this endpoint measurement. All assessment times were included in the final analysis. Failure was defined as a PPV attempt that led to the inability to place the assigned device or inability to achieve 10 consecutive, adequate breaths within 10 min. 

### 2.6. Surveys

We measured perceived confidence using pre- and post-surveys administered to each participant. The surveys utilized a 5-point Likert scale, with 1 representing not confident at all, and 5 representing very confident (Supplements S2 and S3). 

### 2.7. Outcomes and Data Analysis

Outcomes: The primary outcome for this study is time to effective ventilation. The secondary outcome is perceived confidence in using either the LMA or neonatal facemask to provide effective PPV in a manikin or neonate. For this study, Mann Whitney U testing was utilized using an online calculator (www.socscistatistics.com) (accessed on 1 January 2022). The results of the pre- and post-intervention surveys were also analyzed using Mann Whitney U tests.

## 3. Results

We had 32 participants in this study. None of the participants had prior experience with neonatal resuscitation. Sixteen participants were randomized to each of the LMA and neonatal facemask groups. 

### 3.1. Time to Effective Ventilation

Participants randomized to the neonatal facemask-specific learning intervention had a shorter time to effective ventilation than participants randomized to the LMA in a neonatal manikin: 43 (30, 112) seconds vs. 82 (61, 264) seconds, *p* < 0.01 (reported as median and interquartile ranges). In the LMA group, there were two participants that failed to provide effective ventilation during one of their two attempts. There were no failures in the facemask group. 

### 3.2. Reported Confidence

There were no differences in reported confidence for providing adequate ventilation to a neonatal manikin or a neonate prior to the intervention between the two groups (Table 1). However, after the intervention, participants randomized to the neonatal facemask reported higher confidence in providing adequate ventilation to a neonatal manikin than their counterparts randomized to the LMA (5 (4, 5) vs. 4 (4, 4), *p* = 0.03, reported as median (1st interquartile, 3rd interquartile, Table 1)). There was no difference in perceived confidence in providing adequate ventilation to a neonate after the intervention, and there was also no difference in perceived ease of learning the intervention prior to and after the intervention.

## 4. Discussion

This study found that the neonatal facemask with a T-piece resuscitator is superior to the inflatable cuff LMA in providing PPV to a neonatal manikin by healthcare providers with limited neonatal airway experience. Both time to effective ventilation and perceived confidence were higher in the neonatal facemask group. 

Time to achieve 10 adequate consecutive breaths (as an indicator of effective ventilation) was our primary outcome. This measurement was selected as it is a measurement of a physiologic endpoint and assesses not only the ability to place the assigned device correctly but also assesses continued, effective ventilation. Time to effective ventilation was relatively prolonged in both of our study groups. As our definition of this end point required ten consecutive effective breaths, one inadequate tidal volume or device adjustment that affected administration of breaths during the trial negated any prior successful breaths and the count restarted. We preferred a stringent definition of effective ventilation as having consistent effective ventilation, as it may have more clinical relevance, although it may have proved difficult for our participants to provide quick and timely ventilation. 

We intentionally selected an inexperienced population to build a foundation for translating our findings to health care personnel who do not frequently resuscitate neonates. Although the UC Davis Children’s Hospital has a quaternary-level NICU, the community hospitals in our region do not and, as such, likely have practitioners that are not as familiar or adept with neonatal resuscitation. As we continue to think about the LMA as first-line resuscitative therapy for neonatal resuscitation, it becomes necessary to evaluate the ease of use of the LMA and the confidence of providers using the LMA, which was the objective of this study. 

Our study has several limitations. A major limitation of this study was in the use of manikins as a proxy for neonates. Manikins are rigid, produce no oral secretions, and it is possibly easier to create an effective seal on with either a neonatal facemask or LMA than a neonate [19]. The study team attempted to mitigate these differences by using mineral oil for lubrication and instructing participants to look for chest rise and fall, however, due to the nature of the manikin used, we were unable to account for differences in the ability to achieve an effective seal, as we were unable to specifically measure percentage leak around the interfaces. Neonates requiring PPV shortly after birth have airways that can be easily obstructed, owing to poor positioning and immature musculature. This concern of obstruction may be further exacerbated with the use of a neonatal facemask, which is not adequately accounted for in a neonatal manikin, as it is rather rigid. In practice, neonates may benefit more from a supraglottic airway to eliminate the risk of airway obstruction. For these reasons, the manikin is not the ideal model for this study; however, due to the ethics associated with having minimally trained personnel resuscitating neonates that require PPV, the manikin was our most feasible model to evaluate our study question. Additionally, the manikin required a preset tidal volume that needed to be administered prior to study testing. We preset the tidal volume by administering multiple breaths from an NRP-trained physician, as this was the only feasible method of establishing an adequate tidal volume. 

This study also used medical students as our proxy for the unskilled provider, and while this population provides a rich basis for medically knowledgeable participants without much experience in resuscitation, there are some differences in the fund of knowledge that must be considered. Medical professionals providing neonatal resuscitation as a part of their clinical practice have knowledge and familiarity with both intervention devices, which is not necessarily true for medical students. Medical students are familiar with the use of manikins and the facemask due to preexisting requirements of the medical school. All medical students at the UC Davis School of Medicine are required to have up to date Basic Life Support training and certification from the American Heart Association, and thus have been exposed to the use of facemasks in adult and pediatric populations. However, in this study, participants had no prior experience with the use of the LMA, which may have posed a difference in relative confidence and ability in using the LMA versus a neonatal facemask. Of note, there was no statistically significant difference in perceived confidence prior to the administration of the learning intervention. 

The final limitation of our study was that we utilized the cuffed LMA, the LMA device that is most commonly used in the NICU at the UC Davis Children’s Hospital. This may have added an additional step of inflation of the cuff, which would extend the time for LMA placement. Most recent and larger studies of LMA use in neonates have used the i-gel LMA, which is cuffless [5]. Additionally, a study that compared supraglottic airway devices showed that the i-gel LMA has fewer insertion failures and better perceived ease of use than the other supraglottic airway devices trialed [20]. 

In this study, we saw lower confidence in the use of the LMA in ventilating a manikin after the intervention. This finding is consistent with some previous studies [8]. When looking ahead towards implementing the LMA as first-line resuscitative therapy for neonatal resuscitation, we could consider optimizing the learning intervention. To do this, we could implement spaced practice, deliberate practice, and mastery learning [21,22]. Spaced practice would involve multiple sessions and exposures to the LMA over time, whether that be through online video modules or live teaching and practice. Deliberate practice and mastery learning are techniques that give participants discrete goals with ample time and adequate, immediate feedback to achieve them. Continuous practice and defined success with the LMA would also inspire confidence in students and unskilled providers [21,22]. Likely, with increased training and practice time, participants may demonstrate improvement in proficiency and confidence with the use of the LMA. Addressing this potential gap in confidence with additional training will prove to be important when recommending the LMA to new providers who may be unfamiliar with the device. Additionally, studies that use a similar study cohort and different LMAs may find that performance and confidence changes with the different LMAs, which may also inform practice recommendations. 

## 5. Conclusions

In conclusion, pre-clinical medical students demonstrated a shorter time to effective ventilation and reported higher confidence in ventilating a neonatal manikin post-intervention when using a neonatal facemask compared to an LMA. 

Further research is needed to determine if optimization of learning interventions or differences in device used could influence providers’ confidence or performance with an LMA and facemask. We speculate that additional training may be needed for less experienced providers to have ample proficiency and confidence in the use of the LMA. 

In addition, creation of improved neonatal models that replicate the airway challenges that we observe in neonatal resuscitation would allow a more translatable study of the optimal means of providing PPV in neonates.

## Figures and Tables

**Table 1:** Perceived confidence of pre-clinical medical students in performing adequate ventilation with a neonatal face mask and laryngeal mask airway (LMA). The pre- and post-intervention surveys utilized a 5-point Likert scale with 1 representing “Not at all confident” and 5 representing “very confident”. Data presented as median and interquartile ranges.

Parameter	Neonatal Facemask	LMA	*p*-Value
Perceived Confidence in Ventilating a Neonatal Manikin prior to Intervention	1	1	0.81
	(1, 1)	(1, 1)	
Perceived Confidence in Ventilating a Neonate prior to Intervention	1	1	1
	(1, 1)	(1, 1)	
Perceived Confidence in Ventilating a Neonatal Manikin post Intervention	5	4	0.03
	(4, 5)	(4, 4)	
Perceived Confidence in Ventilating a Neonate post Intervention	4	3	0.20
	(3, 4)	(3, 4)	

Data presented as median and interquartile ranges.

## Data Availability

The data presented in this study are available on request from the corresponding author. The data are not publicly available due to maintenance of privacy for our participants.

## Supplementary Materials

The following supporting information can be downloaded at: https://www.mdpi.com/article/10.3390/children10030498/s1, Supplement S1: Flow Diagram of Study Design; Supplement S2: Pre-Participation Survey; Supplement S3: Post-Participation Survey.
