# Supplementary material for: Time to Effective Ventilation in Neonatal Manikins with a Supraglottic Airway vs. a Facemask: A Randomized Controlled Trial
Source: Children (Basel). 2023 Mar 2;10(3):498. doi: 10.3390/children10030498 (PMC10047032; doi:10.3390/children10030498)
Supplement: Supplementary file 1 [file children-10-00498-s001.zip › children-2212016-supplementary.pdf]

Supplement S1: Flow Diagram for Study Design

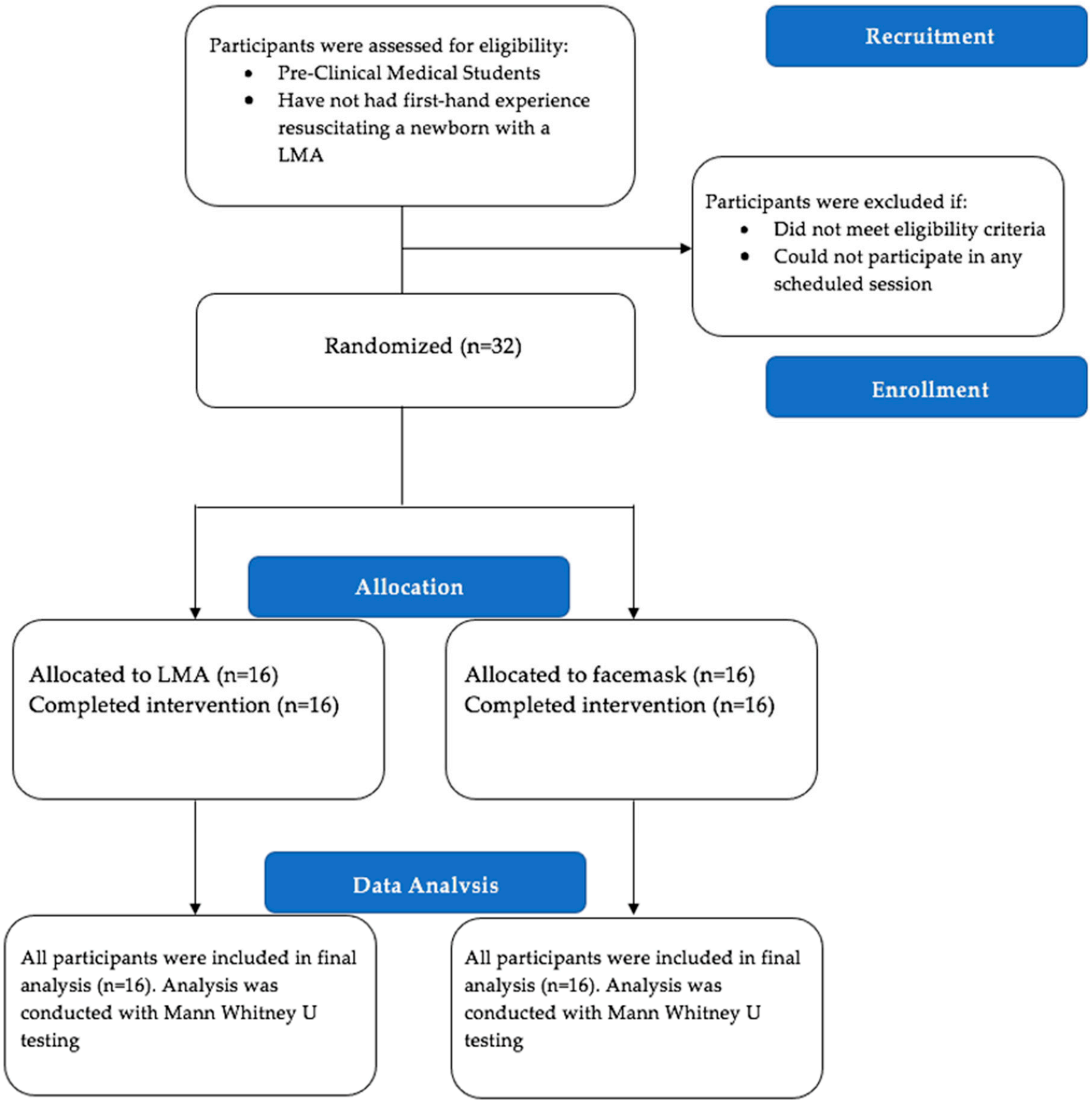

Supplement S2: Pre-Participation survey provided to all participants prior to any administration of study materials.

Study Title: Neonatal Resuscitation with LMAs and Neonatal Facemasks in the hands of unskilled providers
Subject ID:
Intervention Randomized To:

PRE-PARTICIPATION SURVEY
Directions: Please complete this questionnaire BEFORE you begin viewing any training-related material. The questions will have "(LMA/Facemask)" with one intervention circled. Please answer the questions in regards to the circled intervention. Please be as honest as possible and let a member of the present study team know if you have any questions or concerns.

A) Have you previously received any training in neonatal resuscitation?

☐ Yes

☐ No

B) How confident are you in the use of a (LMA/Facemask) in a neonatal manikin?

Not confident at all		Somewhat confident		Very confident
1	2	3	4	5

C) How confident are you in the use of a (LMA/Facemask) in a newborn baby?

Not confident at all		Somewhat confident		Very confident
1	2	3	4	5

D) Rate your perception of ease of learning to use of a (LMA/Facemask) in a neonatal manikin.

Very Difficult	Difficult	Average	Easy	Very easy
1	2	3	4	5

D) Rate your ability to provide effective ventilation during resuscitation in a neonatal manikin with a (LMA/Facemask).

Very Poor	Poor	Average	Good	Very Good
1	2	3	4	5

D) Rate your ability to provide effective ventilation during resuscitation in a newborn with a (LMA/Facemask).

Very Poor	Poor	Average	Good	Very Good
1	2	3	4	5

This is the end of the questionnaire! Thank you for completing it and please make sure to complete the post-participation survey. If you have any questions about the study or this questionnaire, please feel free to contact Nithya at nsivakumar@ucdavis.edu.

Supplement S3: Post-Participation survey provided to all participants after completion of study parameters

Study Title: Neonatal Resuscitation with LMAs and Neonatal Facemasks in the hands of unskilled providers
Subject ID:
Intervention Randomized To:

POST-PARTICIPATION SURVEY
Directions: Please complete this questionnaire AFTER you have completed training and both simulation rounds. The questions will have "(LMA/Facemask)" with one intervention circled. Please answer the questions in regards to the circled intervention.

Please be as honest as possible and let a member of the present study team know if you have any questions or concerns.

A) After this session, how confident are you in the use of a (LMA/Facemask) in a neonatal manikin?

Not confident at all		Somewhat confident		Very confident
1	2	3	4	5

B) After this session, how confident are you in the use of a (LMA/ Facemask) in a newborn baby?

Not confident at all		Somewhat confident		Very confident
1	2	3	4	5

C) Rate your perception of ease of learning to use of a (LMA/ Facemask) in a neonatal manikin after your participation in this session.

Very Difficult	Difficult	Average	Easy	Very easy
1	2	3	4	5

D) Rate your ability to provide effective ventilation during resuscitation in a neonatal manikin with a (LMA/ Facemask).

Very Poor	Poor	Average	Good	Very Good
1	2	3	4	5

E) Rate your ability to provide effective ventilation during resuscitation in a newborn with a (LMA/ Facemask).

Very Poor	Poor	Average	Good	Very Good
1	2	3	4	5

This is the end of the questionnaire! Thank you for completing it and thank you for your participation in this study. If you have any questions about the study or this questionnaire, please feel free to contact Nithya at nsivakumar@ucdavis.edu.
